# Improved oxygenation following methylprednisolone therapy and survival in paediatric acute respiratory distress syndrome

**DOI:** 10.1371/journal.pone.0225737

**Published:** 2019-11-26

**Authors:** Rebecca B. Mitting, Samiran Ray, Michael Raffles, Helen Egan, Paul Goley, Mark Peters, Simon Nadel

**Affiliations:** 1 Paediatric Intensive Care Unit, Imperial College Healthcare NHS Trust, London, United Kingdom; 2 Paediatric and Neonatal Intensive Care Unit, Great Ormond Street Hospital NHS Trust, London, United Kingdom; 3 Respiratory, critical care and anaesthesia section, UCL Great Ormond Street Institute of Child Health, London, United Kingdom; East Carolina University Brody School of Medicine, UNITED STATES

## Abstract

**Background:**

Methylprednisolone remains a commonly used ancillary therapy for paediatric acute respiratory distress syndrome (PARDS), despite a lack of level 1 evidence to justify its use. When planning prospective trials it is useful to define response to therapy and to identify if there is differential response in certain patients, i.e. existence of ‘responders’ and ‘non responders’ to therapy. This retrospective, observational study carried out in 2 tertiary referral paediatric intensive care units aims to characterize the change in Oxygen Saturation Index, following the administration of low dose methylprednisolone in a cohort of patients with PARDS, to identify what proportion of children treated demonstrated response, whether any particular characteristics predict response to therapy, and to determine if a positive response to corticosteroids is associated with reduced Paediatric Intensive Care Unit mortality.

**Methods:**

All patients who received prolonged, low dose, IV methylprednisolone for the specific indication of PARDS over a 5-year period (2011–2016) who met the PALICC criteria for PARDS at the time of commencement of steroid were included (n = 78).OSI was calculated four times per day from admission until discharge from PICU (or death). Patients with ≥20% improvement in their mean daily OSI within 72 hours of commencement of methylprednisolone were classified as ‘responders’. Primary outcome measure was survival to PICU discharge.

**Results:**

Mean OSI of the cohort increased until the day of steroid commencement then improved thereafter. 59% of patients demonstrated a response to steroids. Baseline characteristics were similar between responders and non-responders. Survival to PICU discharge was significantly higher in ‘responders’ (74% vs 41% OR 4.14(1.57–10.87) p = 0.004). On multivariable analysis using likely confounders, response to steroid was an independent predictor of survival to PICU discharge (p = 0.002). Non-responders died earlier after steroid administration than responders (p = 0.003).

**Conclusions:**

An improvement in OSI was observed in 60% of patients following initiation of low dose methylprednisolone therapy in this cohort of patients with PARDS. Baseline characteristics fail to demonstrate a difference between responders and non-responders. A 20% improvement in OSI after commencement of methylprednisolone was independently predictive of survival, Prospective trials are needed to establish if there is a benefit from this therapy.

## Background

Corticosteroids are a widely used ancillary therapy for paediatric acute respiratory distress syndrome (PARDS), despite a lack of robust evidence of benefit [1, 2]. The 2015 Pediatric Acute Lung Injury Consensus Conference (PALICC) recommended that ‘further study should focus on specific patient populations that are likely to benefit from corticosteroid therapy and specific dosing and delivery regimens.’ [3] Data from adult patients are conflicting. A 2016 meta-analysis of corticosteroid trials in ARDS showed reduced hospital mortality and increased ventilator free days (VFDs) [4]. However, corticosteroids late in the course of ARDS may be harmful. The ARDSnet Late Steroid Rescue Study observed an increase in serious infections and myopathy with steroid use after 14 days of ARDS, without survival benefit [5]. Targeting patients in the fibroproliferative phase of ARDS is a potential strategy to improve risk/benefit ratio [6]. This risk/benefit ratio may be different in children, in whom nosocomial infections are less common. [7].

Paediatric data to date are limited to case series and an observational study [1,8]. The difficulties with designing a randomised controlled trial (RCT) are manifold, owing to low case frequency, and the dominant impact of comorbidity on mortality and length of Paediatric Intensive Care Unit (PICU) stay [2,9]. Further heterogeneity arises from distinct sub-types of ARDS: ‘direct’ (pulmonary) and ‘indirect’ (non-pulmonary) [10,11].

In view of the heterogeneity of the PARDS population, an important step in the design of future prospective trials may involve the identification of patients who are physiological ‘responders’ to a therapy, as well as definition of a clinically significant response to therapy [12,13]. This is especially important regarding treatments such as corticosteroid therapy where the possibility of harm has been identified [1]. The harm done to non-responding patients may counteract the benefit of a therapy for responding patients, resulting in a ‘negative trial’ [12,13].

### Aims

1. To identify if there is improvement in the mean OSI of a cohort of patients with severe PARDS following introduction of IV methylprednisolone.
2. To identify what proportion of patients treated with IV methylprednislone demonstrate a 20% improvement in mean daily OSI following initiation of therapy.
3. To identify if there are differing characteristics between ‘responders’ and ‘non responders’ to therapy.
4. To establish if a response to steroids is associated with reduced mortality. (We have learnt from studies looking at use of inhaled nitric oxide for hypoxic respiratory failure that an improvement in oxygenation does not always translate to reduction in mortality [14]).

We hypothesized that mean OSI of the cohort would improve following the administration of corticosteroids to patients with severe PARDS, and that a significant early improvement (in <72 hours) in gas exchange (≥20% fall in OSI) following administration of corticosteroids for PARDS would be associated with PICU survival.

## Methods

### Population

This was a retrospective observational study conducted at two paediatric intensive care units in two separate hospitals, within Imperial College Healthcare NHS Trust (ICH), and Great Ormond Street Hospital (GOSH) in London, United Kingdom. The study was approved as a clinical audit by Imperial College Healthcare NHS Trust Information Governance department (registration number 974626), and by the Clinical Audit Department at Great Ormond Street Hospital (registration number 2385) and no patient identifiable data are reported, therefore individual patient consent was not sought. In both institutions steroids are used in accordance with the ARDSnet LaSRS trial treatment arm protocol, a loading dose of 2mg/kg of methylprednisolone followed by 2mg/kg/day in divided doses for 14 days or until weaned from mechanical ventilation [5]. Permissive hypercapnia (targeting pH ≥ 7.25) is practiced for patients with lung disease (excepting patients with pulmonary hypertension and raised intracranial pressure) and low tidal volume ventilation (5-8ml/kg) is practiced for all patients with PARDS. Inhaled nitric oxide is not recommended for routine use but employed as a rescue therapy at the discretion of the treating clinician. Both centres use a minimum PEEP of 5cm H_2_O in all invasively ventilated patients and use High Frequency Oscillatory ventilation and prone positioning at the discretion of the treating clinician. There is a shared pathway for referral for extra-corporeal membrane oxygenation (ECMO) between both centres.

All patients who had been prescribed methylprednisolone for the specific indication of PARDS on PICU (between 1st Jan 2011 to 31^st^ Dec 2016 at ICH and 1^st^ Apr 2012 to 31^st^ Dec 2016 at GOSH) were identified from the electronic health record system (Philips Intellivue Critical Care and Anesthesia^™^, Philips Electronics, Amsterdam, the Netherlands). The differing start dates for data collection is owing to the later introduction of the electronic health record system at Great Ormond Street Hospital. Patients were excluded if they did not meet the PALICC criteria for definition of PARDS (within 7 days of an insult, symptoms not fully explained by cardiac failure or fluid overload, new infiltrates on chest X-Ray, OI > 8 (or OSI >7.5) on the day of administration of the first dose of steroid for PARDS). They were also excluded if the primary indication for the administration of the steroid was not PARDS following case-note review, or if they had documented pulmonary pathology known to be responsive to corticosteroid therapy. This is a retrospective cohort study and therefore a power calculation was not performed.

### Data collection

Data recorded included: age, gender, diagnosis at admission; comorbidities; methylprednisolone dose; days of ventilation prior to initiation of therapy; duration of therapy; use of inhaled nitric oxide (iNO) prior to steroid initiation; duration of invasive mechanical ventilation (IMV); exposure to continuous neuromuscular blockade prior to steroid initiation; prone positioning prior to steroid initiation; length of PICU stay and survival to PICU discharge. Cumulative fluid balance was calculated by recording the cumulative fluid balance at 0700 hours on the day of starting steroids, and subtracting the cumulative fluid balance at the time of initiation of invasive mechanical ventilation. PIM-3 score was recorded at admission to ICU, and paediatric logistic organ dysfunction (PELOD) score was recorded at the point of commencement of methylprednisolone [15]. Prior steroid exposure was defined as having been prescribed a corticosteroid for any indication, prior to the methylprednisolone course commencement for PARDS during this PICU admission. Immune compromise was defined as the presence of a congenital immune deficiency, an immunocompromising diagnosis (haematopoetic stem cell transplant, haematological malignancy) or treatment with immunosuppressive chemotherapy. All infants born before 37 weeks completed gestation were recorded as preterm. In patients who did not survive, number of days between steroid commencement and day of death was recorded.

The PALICC definition of PARDS uses oxygenation index (OI) rather than PaO2/FiO2 ratio as the respiratory criterion for disease severity [16]. Critically ill children are less likely to have arterial blood gas measurements than adult patients, owing to technical difficulties. Oxygen Saturation Index (OSI), using haemoglobin oxygen saturation determined by pulse oximetry (SpO2), is recommended when OI is not available [16]. OSI has a linear relationship to OI when saturations are < 97% [17]. It has been shown to predict clinical outcomes in both adult and paediatric patients with ARDS [18, 19]

In retrospective analysis of data, there are difficulties with using OI as a marker of illness progression or improvement, as indwelling arterial catheters are often removed when there is clinical improvement, leading to bias. There is also a high degree of variability, and the limited number of values in a 24-hour period leads to a reduction in accuracy of analysis. Blood gases are often taken following a clinical change and may not accurately, therefore, reflect the overall trend in the patients’ clinical condition. The use of OSI overcomes these problems, as it can be measured until a patient is separated from invasive mechanical ventilation and in the majority of patients, is measured and recorded hourly.

OI was calculated daily for all patients from the first arterial blood gas after midnight for all admitted days. OSI ([FiO_2_ × Mean airway pressure × 100]/SpO_2_) was calculated at four time points, 0000, 0600, 1200 and 1800, daily for the admission, ensuring that only SpO_2_ values between 80%-97% were used for analysis [17]. The day of commencement of methylprednisolone for PARDS is referred to as day 0 for description and analysis, with positive integers used to describe the days following steroid commencement (day 1,2,3 etc) and negative integers for days prior to steroid commencement.

A ≥20% improvement in mean daily OSI, within 72 hours of day 0 was determined a priori to be response to methylprednisolone. We selected this definition based on previous studies which had used OI/OSI as an outcome measure [20, 21].

Complications were defined as a requirement for antihypertensive therapy, diagnosis of a new infection, or a new requirement for insulin therapy.

### Statistical analysis

Descriptive statistics included mean and standard deviation for normally distributed and median and interquartile range for skewed data for non-parametric data. Normality of continuous values were tested using the Kolmogorov-Smirnov test. Continuous variables were compared using t-test if normal or the Mann-Whitney test if non-normal; categorical variables were compared using Fisher’s exact t-test. A p-value of <0.05 was considered to be statistically significant.

To characterise the change in OSI over time we used a multi-level linear regression model with OSI as the dependent variable, days post admission as the fixed effect variable, and patient identifier as the random effect variable. This accounted for individual patient variation in response and missing values for time-points. OSI is presented log transformed for normalcy.

The odds and likelihood ratios of survival following response to steroids are presented, alongside the predictive value of response to steroids for survival.

Multivariable logistic regression was performed to test for independent association between response to steroids and PICU mortality and included likely confounders: immune compromise; prior steroid exposure; exposure to neuromuscular blockade; prone positioning; cumulative fluid balance on day 0 and PELOD score on day 0.

A Cox proportional hazards model was used to analyse the difference in the time to death between responders and non-responders, using the same covariates as above.

All data were analysed using Microsoft Excel^™^ (Microsoft Corp., Redmond, WA) and r^™^ (www.r-cran.org).

## Results

**Fig 1:**
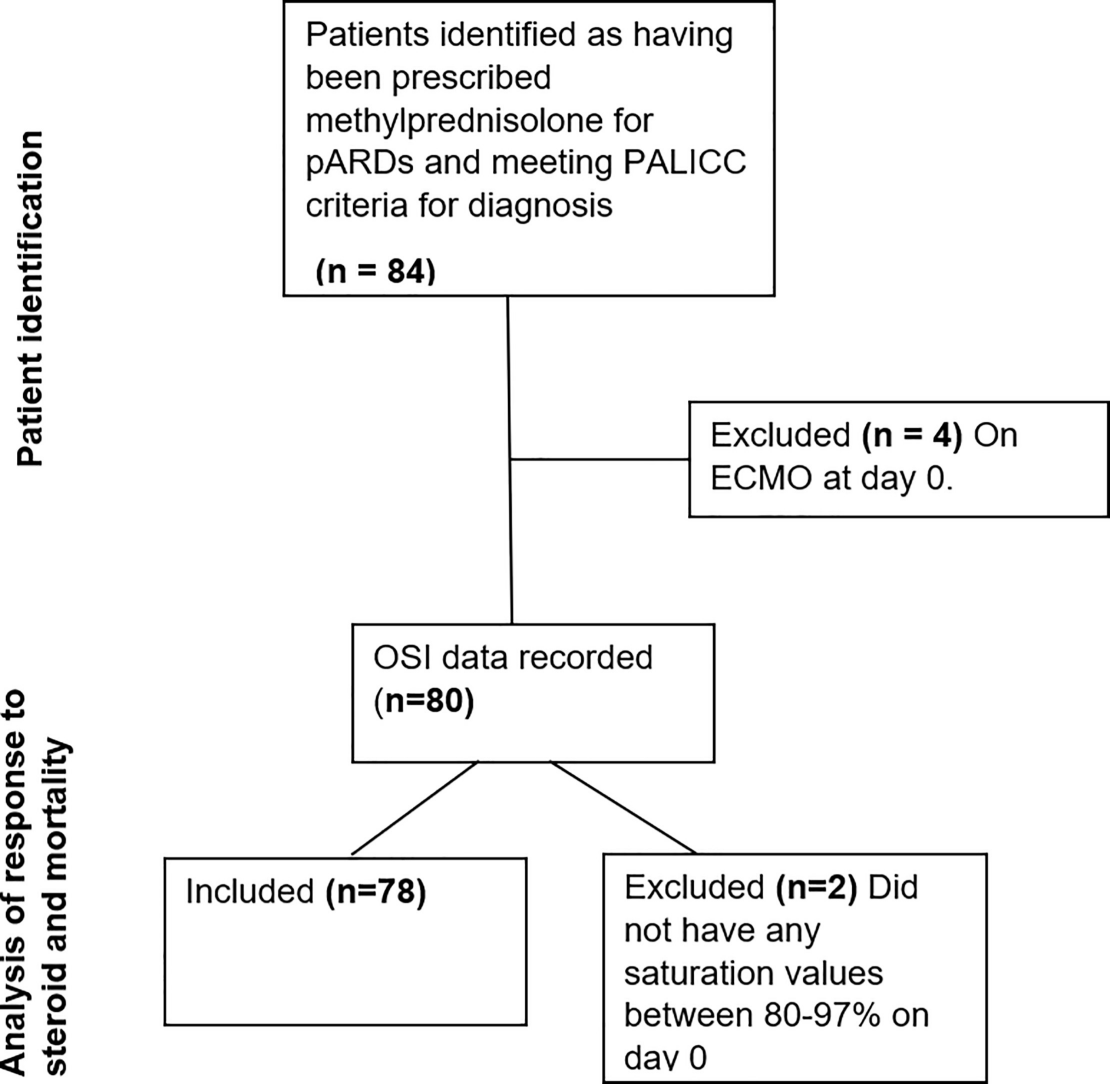
Patient selection and inclusion.

Between January 2012 (2011 at Imperial College Healthcare NHS Trust) and December 2016, from a total of 6470 PICU admissions, 84 patients met inclusion criteria. Four patients were excluded from analysis as they were receiving extra-corporal membrane oxygenation (ECMO) at the time of commencement of methylprednisolone (four other children received ECMO distant to the commencement of steroids and were therefore included in the analysis). Two patients had no haemoglobin oxygen saturation measurements between 80–97% on the day of steroid commencement, meaning that OSI could not be calculated. The remaining patients were included (n = 78) (Fig 1)

Median age was 9 months (IQR 4–27). There were 35 (45%) female patients. Sixty-five (83%) patients had ‘direct’ PARDS, and the most common trigger was pneumonia (n = 53). Eleven (14%) patients were immune compromised and 36 patients (46%) had been born preterm. Fifty (64%) patients received inhaled nitric oxide during their PICU stay. 44 of these 50 patients received inhaled nitric oxide prior to commencement of steroids.

Forty-seven (60%) patients survived to ICU discharge. Median PELOD score at commencement of steroids was 10 (IQR 1–11). Median PIM-3 score was 0.06 (IQR 0.03–0.09). Median OI (from blood gas analysis of 63 patients who had an indwelling arterial catheter) on day 0 was 19.5. The median duration of IV methylprednislone therapy was 11 days. Sixteen (21%) patients had prior steroid exposure during this PICU stay. Ninety four percent (n = 75) of patients had received continuous neuromuscular blockade prior to initiation of steroid therapy and 53% (n = 42). of patients had been nursed prone (n = 42).

Methylprednisolone was commenced on day 9 (median, IQR 6–13) of admission, meaning that for most patients ‘steroid day 0’ of this study falls within their second week of invasive mechanical ventilation for PARDS.

**Fig 2:**
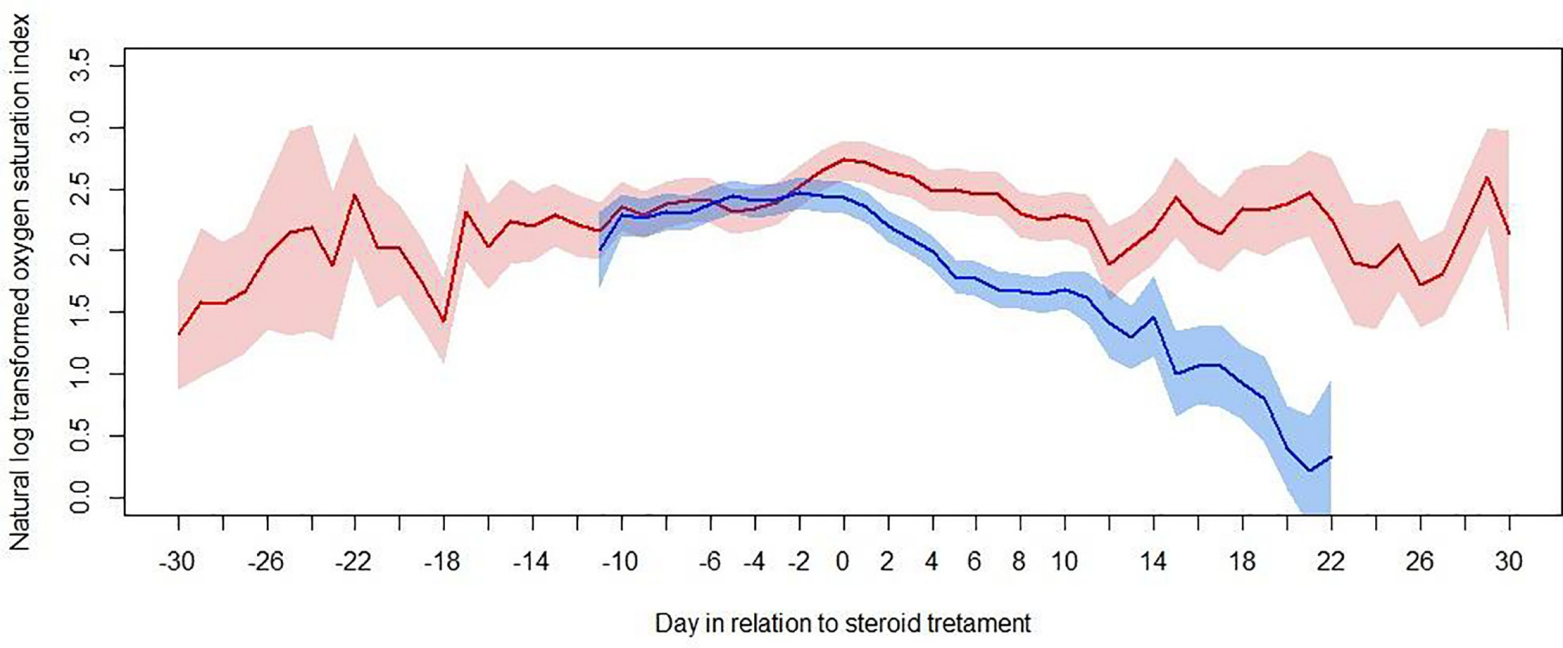
In Oxygen Saturation Index before and after steroid therapy in survivors and non- survivors. Mean and 95% CI calculated using a multi-level linear regression model with OSI (log transformed) as the dependent variable, days post admission as the fixed effect variable, and patient identifier as the random effect variable.

OSI data were analysed for 78 patients. All SpO2 values <80% or >97% were excluded (n = 319). Excluding values that fell outside of the SpO_2_ range 80–97% there were a total of 4393 values. Median OSI on day 0 was 13.4 (IQR 9.9–19.4). A multi-level linear regression model was used to characterise the association between OSI and day in relation to commencement of steroid, with patients separated according to survival status (Fig 2).

Forty-six (59%) patients had a 20% or greater improvement in mean daily OSI within 72 hours of steroid commencement (‘responders’). Baseline characteristics, including age, PARDS aetiology, frequency of immune compromise, use of neuromuscular blockade, proning, frequency of prematurity and severity of illness (PIM-3, PELOD, OSI at commencement of steroid) were similar between ‘responders’ and ‘non-responders’ (Table 1). Responders and non-responders also had similar frequency of prior steroid exposure, and number of days of ventilation prior to commencement of steroid, as well as similar cumulative fluid balance at the time of steroid commencement.

**Table 1:** Characteristics of paediatric intensive care unit patients by oxygenation response to intravenous methylprednisolone.

Characteristic	Responders(n = 46)	Non-responders(n = 32)	p-value
Age in months, median(IQR)	9.0 (5.0–22.0)	13.0 (6.8–36.0)	0.22
Unit–GOSH n (%)	15 (33)	9 (28)	0.80
Unit ICH n (%)	31 (67)	23 (72)	
Diagnosis pneumonia n (%)	32 (70)	24 (75)	0.51
Diagnosis sepsis n (%)	5 (11)	1 (3)	
Diagnosis post surgery n(%)	3 (7)	4 (9)	
Diagnosis other n (%)	6 (13)	3 (7)	
Immune compromise n (%)	8 (17)	4 (9)	0.75
Premature n (%)	19 (41)	17 (53)	0.36
Direct injury n (%)	38 (83)	27 (84)	1.00
Infection n (%)	36 (78)	24 (75)	0.79
Prior steroid exposure n (%)	9 (20)	8 (25)	0.78
Inhaled nitric oxide use prior toinitiation of steroid n (%)	22 (48)	22 (69)	0.1
PIM-3, median (IQR)	0.067 (0.27–0.12)	0.05 (0.27–0.08)	0.37
Oxygenation Saturation Index on day of start of steroids median(IQR)	13.1 (9.4–18.4)	14.0 (10.2–20.5)	0.65
Days ventilated prior to start of steroids median(IQR)	9.5 (6.0–12.0)	9.0 (6.0–14.0)	0.51
PELOD on day of starting steroid median(IQR)	10.0 (1.0–11.0)	10.5 (1.0–11.3)	0.74
Neuro-muscular blockade prior to steroids (%)	44 (96)	30 (94)	1
Proning prior to steroids (%)	23 (50)	19 (59)	0.49
Fluid balance on day of starting steroids, ml/kg Median (IQR)	90.6 (25.8–183.7)	84.7 (20.1–163.1)	0.87
Survival at ICU discharge n (%)	34 (74)	13 (41)	**0.005**

Thirty-four (74%) of 46 ‘responders’ and 13/32 (41%) ‘non-responders’ survived to PICU discharge (OR 4.14, 1.57–10.87 p = 0.004). The positive likelihood ratio was 1.87 (1.16–3.01), with a positive predictive value 74% (64–82) and negative predictive value of 59% (46–72).

As well as response to methylprednisolone, admitting unit, prior steroid exposure, PELOD score day 0, OSI day 0 and *lower* PIM-3 score at admission to PICU were associated with PICU mortality in univariate analysis. (Table 2).

**Table 2:** Characteristics of paediatric acute respiratory distress syndrome patients by paediatric intensive care unit survival status.

Characteristic	Survived (n = 47)	Died (n = 31)	p-value
Age in months, median(IQR)	11.0 (6.0–22.0)	10.0 (6.5–36.0)	0.63
Unit GOSH n (%)	10 (21)	14 (45)	**0.04**
Unit ICH n (%)	37 (79)	17 (55)	
Diagnosis pneumonia n (%)	37 (79)	19 (61)	0.27
Diagnosis sepsis n (%)	3 (6)	3 (10)	
Diagnosis post surgery n(%)	4 (9)	3 (10)	
Diagnosis other n(%)	3 (6)	6 (19)	
Immune compromise n (%)	5 (11)	7 (23)	0.20
Premature n (%)	23 (49)	13 (42)	0.64
Direct injury n (%)	40 (85)	25 (81)	0.76
Infection n (%)	39 (83)	21 (68)	0.17
Prior steroid exposure n (%)	6 (13)	11 (36)	**0.02**
Inhaled nitric oxide use prior to initiation of steroid n (%)	28 (60)	16 (52)	0.49
PIM-3, median(IQR) n (%)	0.07 (0.04–0.10)	0.04 (0.02–0.08)	**0.02**
Oxygenation Saturation Index on day of start of steroids, median(IQR)	12.3 (8.8–15.6)	15.3 (12.0–21.4)	**0.01**
Days ventilated prior to start of steroids, median(IQR)	9.0 (6.0–13.0)	10.0 (6.0–12.5)	0.72
PELOD on day of starting steroid, median(IQR)	2.0 (1.0–11.0)	11.0 (10.0–12.5)	**0.02**
Neuro-muscular blockade prior to steroids (%)	44 (94)	30 (97)	1
Proning prior to steroids (%)	29 (62)	18 (58)	0.11
Fluid balance on day of starting steroids, ml/kg Median (IQR)	90.6 (25.4–163.9)	84.7 (20.5–168.6)	0.78
Response to steroids (≥ = 20% decrease in oxygenation index) n (%)	34 (72)	12 (39)	**0.004**

**Fig 3:**
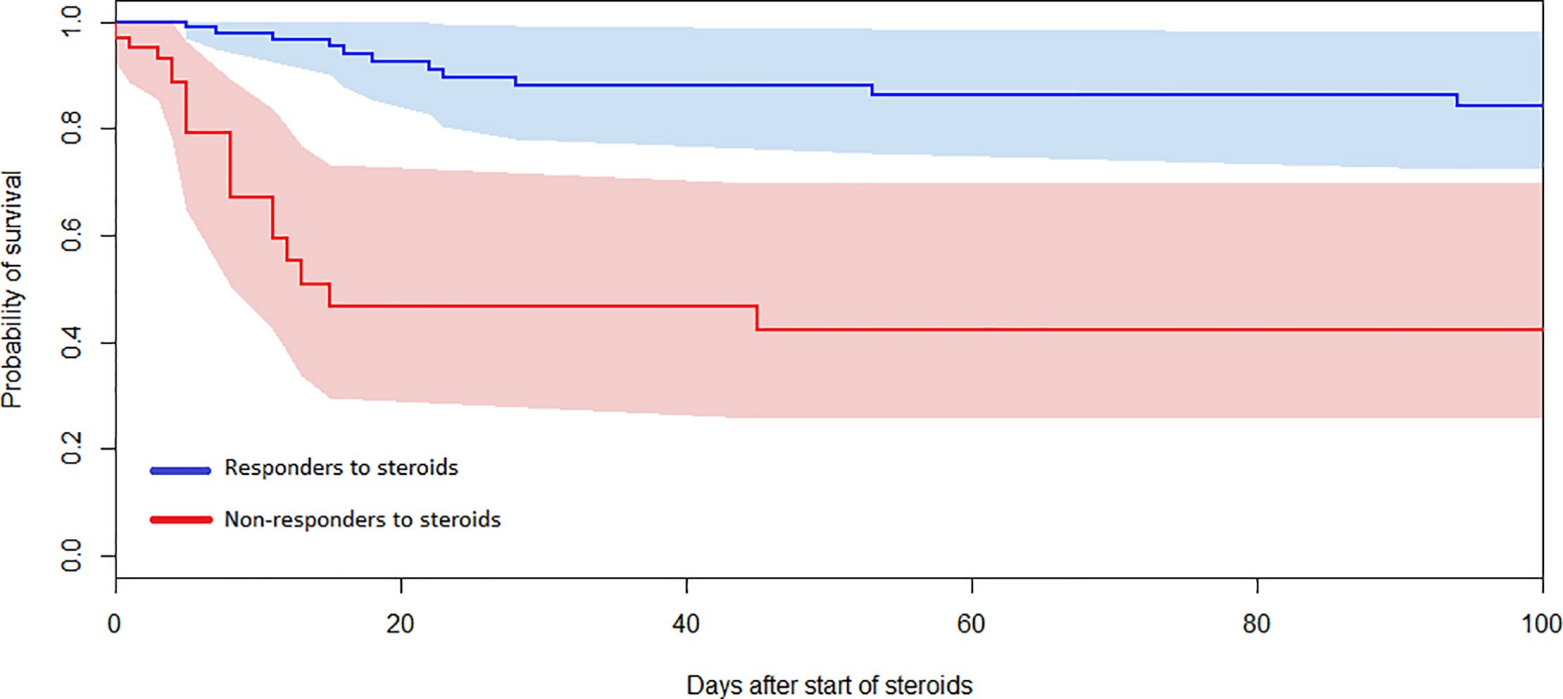
Kaplan-Meier plot of all-cause mortality in days after commencement of steroids and 95% confidence interval (shaded areas) by responder / non-responder to steroid status. The analysis was adjusted for age, PIM-3 score, PELOD, admitting unit, prior steroid use, immune compromise, prematurity, direct/indirect PARDS phenotype (direct/indirect) and infection/non-infection as independent variables in a Cox proportional hazard model, and demonstrates that death occurred earlier in ‘non-responders to steroid therapy (p = 0.003).

On multivariable analysis using likely confounders (organ dysfunction score, immune compromise, neuromuscular blockade, being nursed prone and fluid balance at commencement of steroid) response to steroid was an independent predictor of survival to PICU discharge (p = 0.005)). Having been nursed prone prior to initiation of steroid was also independently predictive of survival (p = 0.04) High PELOD score was associated with lower rates of survival p = 0.048) (Table 3). Using a Cox proportional hazards model with PELOD score and immune compromise, the time to death was found to be significantly different for responders and non-responders, with non-responders dying earlier (p = 0.003) (Fig 3).

**Table 3:** Multivariable logistic regression testing response to steroid and likely con-founders as independent predictors of PICU mortality.

Characteristic	odds ratio of survival	95% CI	p- value
Response to steroids	5.46	1.78–19.01	**0.005**
Use of neuro-muscular blockade	0.42	0.02–4.31	0.49
Use of proning	3.20	1.05–10.80	0.045
Fluid balance/weight on day of starting steroid	1.00	0.99–1.00	0.26
PELOD score	0.92	0.85–1.00	**0.04**
Immune compromise	0.35	0.06–1.90	0.22

A new infection was diagnosed in 30/78 (38%) patients after the commencement of steroids. Five (6%) patients had hypertension requiring oral anti-hypertensive therapy and 15(19%) patients had new onset hyperglycaemia requiring treatment with an insulin infusion for a median duration of 4 days.

## Discussion

In this retrospective, two-centre, observational study, we describe the change in mean OSI in a cohort of patients following the administration of prolonged, low dose methylprednisolone in this cohort of patients with PARDS.

Fig 2 demonstrates that the mean adjusted OSI for the cohort was increasing until the day of commencement of steroid, but after day 0, the mean OSI improves. The gradient of improvement is steeper for surviving patients. Whilst one cannot infer causality from this figure, a temporal association is noted between commencement of steroid and a change in average illness trajectory.

We have demonstrated that 60% of the cohort had an improvement in OSI following administration of corticosteroid therapy. Having a positive response to therapy was associated with improved mortality, and a non-response to steroid can be used to predict likelihood of survival. This new knowledge may be important to consider when designing future prospective trials, as the knowledge that some children will be physiological ‘non-responders’ reduces the chances of a positive trial. It is therefore also an important negative finding of this study that we have not identified any differences between ‘responders’ and ‘non-responders’. The association of steroid responsiveness with survival also helps to confirm that improvement in OSI of 20% is a clinically significant end point to consider.

Non-responders to steroids who died, did so relatively soon after the start of steroids. In comparison ‘responders’ who die tend to die later (Fig 3). This may reflect differences in mode of death: non-responders may die due to irreversible hypoxia, while responders die later due to other co-morbidities. We did not find any differences in the baseline characteristics between ‘responders’ and ‘non-responders’ to steroids. Therefore, predicting response to steroids based on these data for enrichment of a trial population cannot be proposed. On a post hoc analysis of OSI in non-surviving patients, 85% of non-responders and 50% of responders had an OSI of > 12.3 at the time of death, and therefore were more likely to meet the oxygenation criteria for severe PARDS (OR 0.18, 95% CI 0.03–0.04 p = 0.04) when they died, again supporting the hypothesis that non-responders died from refractory hypoxic respiratory failure, and responders died from co-morbidity.

There is a large difference in baseline PELOD score between surviving and non-surviving patients in this study, which is not altogether surprising in view of the obvious association between multi-organ failure and mortality in critically ill patients. There is, however, no difference in PELOD score between ‘responders’ and ‘non-responders’ at the time of commencement of steroid. It may be hypothesised from this data that whilst steroids improve oxygenation, that their side effects are deleterious to patients with multi-organ failure. There is also a significant difference in OSI at the time of commencement of steroids between surviving and non-surviving patients, despite no difference in OSI between responders and non-responders to steroid therapy. This may suggest that, if possible, commencement of steroids at a point where OSI is lower, potentially earlier in the course of the illness, may be more likely to convey a survival benefit if one is to be found. In view of this it may be that future trial use PELOD score and OSI as stratification or minimisation criteria. On post hoc analysis, including OSI at day 0 in a multivariable analysis does reduce the significance of the effect of both PELOD score and proning prior to steroids (S1 Table) but is not in itself independently predictive of mortality.

It is also noted that having been nursed prone prior to commencement of steroid therapy was independently associated with survival. This concurs with adult data which describes mortality benefit with nursing prone for severe ARDS patients [22] and it is noteworthy as it is unknown whether this benefit is certain in the paediatric population who often also receive high frequency oscillatory ventilation.

The largest prior study of corticosteroid use in PARDS was an observational study of 283 patients with PARDS, which found a reduction in VFD with corticosteroid use >24 hours [1]. This study differs from our work in several respects. The patients had a very high frequency of corticosteroid exposure, most commonly on the first day that they met the criteria for PARDS (60% of patients receiving > 24 hours of corticosteroid therapy). Prior to instituting methylprednisolone therapy only 1 in 5 of patients in our cohort had corticosteroid exposure, and methylprednisolone was commenced on a median of day 9 of ventilation. This is relevant when considering evidence for steroid use in the fibroproliferative phase of PARDS [6], or that steroid therapy should be prolonged to avoid a rebound effect [23]. Yeyha et al describe a median total dose of 8mg/kg methylprednisolone equivalent, which is a much lower dose/duration than the 2mg/kg/day typically used in our patients and the ARDSnet protocol [1, 5].

Yehya et al excluded patients with pre-existing respiratory disease from the analysis. This may be beneficial—excluding other conditions such as chronic lung disease of prematurity and reactive airways disease, in which there is clearer evidence of the benefit of steroids [24]. However, in practice this is difficult, as the PICU population is heterogenous, and increasingly a large proportion of children have chronic comorbidities [25]. Many of these children will be pre-disposed to respiratory disease due to risk of gastro-oesophageal aspiration and other susceptibility to infections. Ex-premature children make up a large proportion of patients on PICU with respiratory failure [26], and therefore it is unlikely to be feasible to design a trial which excludes them. In 2010 the PALISI group investigated the feasibility of performing randomised controlled trials for therapeutic interventions in PARDS and estimated that 60 centres may be needed to enrol sufficient numbers of patients. This difficulty is highlighted in our results, finding only 78 patients in 5 years between 2 centres [2]. It is a limitation of this study that it is not possible to achieve complete certainty that patients did not have a lung pathology sensitive to corticosteroid therapy as lung biopsy was not performed.

In 2017 the Society of Critical Care Medicine and European Society of Intensive Care Medicine and European Society of Intensive Care Medicine Guideline was published which suggests the use of IV methylprednisolone in patients with *early* ARDS, within 14 days of onset [27]. We therefore also performed a post hoc multivariable analysis including days of invasive mechanical ventilation prior to commencement of steroid (S1 Table) which found that this did not have a significant effect on survival.

Our study was observational. No causality can be ascribed to the association of a response to methylprednisolone with survival. There is a high level of variability in the reported mortality of PARDS [28,29] ranging from 11.3% in a study including children with acute lung injury (ALI) [30] to 76.5% in a study of children who have undergone haematopoietic stem cell transplant [31]. This is likely owing to the dominant influence of co-morbidity on outcome, with immune compromised children experiencing high mortality. We found a significant difference in the mortality between the 2 PICUs participating in this study, which may be explained by differing patterns of comorbidity in the populations served by the 2 units, not accounted for by the PIM score. It is a further limitation of the study that we were unable to record exact mode of death for all patients owing to the retrospective nature of the review. This means that we cannot exclude that some patients had intensive care therapy withdrawn on the basis of a lack of response to steroid therapy. It is also a limitation of our study that, owing to identification of patients via a database of patients who had been prescribed IV methylprednisolone, we were unable to identify a control group who did not receive corticosteroids for their PARDS.

More sophisticated analysis such as propensity score matching for response to steroids could not be undertaken in this study owing to the small numbers. It is arguable that the association between improved oxygenation and survival is intuitive: in adults, however, improvement in oxygenation in the first 2 days of ARDS has been shown to have limited value in predicting mortality [32].

Regarding the use of OSI in comparison to OI, we carried out a post hoc analysis matching OI and OSI for the patients in whom indwelling arterial catheter was present to enable measurement of PaO2 to confirm correlation between OI and OSI for the cohort. Data were available for 75 patients at some point during invasive mechanical ventilation and there were 900 OI values which could be compared directly with an OSI value. Spearman rank r-squared = 0.77. (S1 Fig).

This observational study demonstrates a pressing need for new research into the use of corticosteroids in PARDS, as it raises the possibility of a mortality benefit with response to prolonged low dose methylprednisolone therapy in this group of patients with high mortality and healthcare costs. We have, however, demonstrated an a high burden of adverse effects, as well as high mortality in children with multi-organ failure, and previous studies have shown increased morbidity with indiscriminate use of corticosteroids [1]. A recent pilot study has demonstrated feasibility of an RCT, although this would not be without challenges. An alternative being a large-scale pragmatic RCT, or multi-centre comparative effectiveness study [33]. The upcoming PROSpect trial may provide a platform upon which a trial of corticosteroid use in PARDS could be built, and it is important to consider the presence of ‘responders’ and ‘non-responders’ in the design, as if it is the case that ‘non-responders’ experience harm from the therapy, this may produce a ‘negative’ trial of a treatment with the potential to benefit some patients. [34].

## Conclusion

An improvement in OSI was observed following initiation of low dose methylprednisolone therapy in 59% of this cohort of patients with severe PARDS. A 20% improvement in OSI following methylprednisolone was independently predictive of survival, suggesting that this is a clinically significant physiological response to therapy. Baseline characteristics failed to demonstrate a difference between responders and non-responders.

## Supporting information

S1 Checklist STROBE checklist for study. (DOC)

S1 Fig Correlation of oxygenation index with Oxygen Saturation Index measurements taken at the same time points for all patients. (JPG)

S1 Table Multivariable logistic regression testing response to steroid and likely con-founders (including OSI at day 0) as independent predictors of PICU mortality. (DOCX)

S2 Table Multivariable logistic regression testing response to steroid and likely con-founders (including day of ventilation commenced on steroid) as independent predictors of PICU mortality. (DOCX)

S1 Dataset Full dataset for study. (XLSX)

## Abbreviations

PARDS: Paediatric acute respiratory distress syndrome
OSI: Oxygen Saturation Index
PICU: Paediatric Intensive Care Unit
ECMO: Extra-corporeal membrane oxygenation
PELOD: Pediatric logistic organ dysfunction
iNO: Inhaled Nitric Oxide
VFDs: Ventilator-free days
PALICC: Pediatric Acute Lung Injury Consensus Conference
SpO2: Haemoglobin oxygen saturation determined by pulse oximetry
ALI: Acute lung injury
RCT: Randomised, controlled trial
