# Supplementary figures and images for: Improved oxygenation following methylprednisolone therapy and survival in paediatric acute respiratory distress syndrome
Source: PLoS One. 2019 Nov 26;14(11):e0225737. doi: 10.1371/journal.pone.0225737 (PMC6879165; doi:10.1371/journal.pone.0225737)

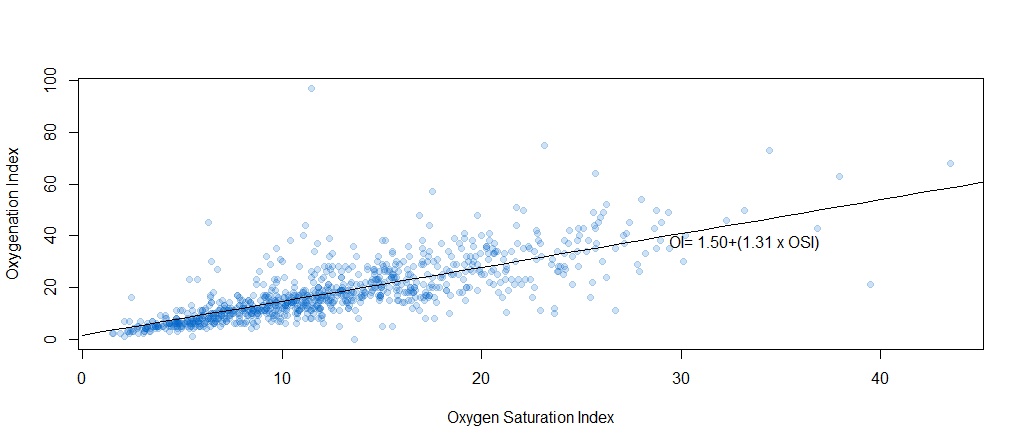

Supplement: S1 Fig — (JPG) [file pone.0225737.s002.jpg]
