# Supplementary material for: Improved oxygenation following methylprednisolone therapy and survival in paediatric acute respiratory distress syndrome
Source: PLoS One. 2019 Nov 26;14(11):e0225737. doi: 10.1371/journal.pone.0225737 (PMC6879165; doi:10.1371/journal.pone.0225737)
Supplement: S1 Table — (DOCX) [file pone.0225737.s003.docx]

| **Characteristic** | **odds ratio of survival** | **95% CI** | **p- value** |
| --- | --- | --- | --- |
| Response to steroids | 5.13 | 1.65-17.88 | 0.006 |
| Use of neuro-muscular blockade | 0.44 | 0.02-4.49 | 0.52 |
| Use of proning | 3.93 | 1.20-14.36 | 0.03 |
| Fluid balance/weight on day of starting steroid | 1.00 | 0.99-1.00 | 0.38 |
| PELOD score | 0.92 | 0.85-1.00 | 0.05 |
| Immune compromise | 0.42 | 0.07-2.59 | 0.33 |
| Day of mechanical ventilation when steroids started | 0.95 | 0.84-1.05 | 0.34 |
