# Supplementary material for: Improved oxygenation following methylprednisolone therapy and survival in paediatric acute respiratory distress syndrome
Source: PLoS One. 2019 Nov 26;14(11):e0225737. doi: 10.1371/journal.pone.0225737 (PMC6879165; doi:10.1371/journal.pone.0225737)
Supplement: S2 Table — (DOCX) [file pone.0225737.s004.docx]

| **Characteristic** | **odds ratio of survival** | **95% CI** | **p- value** |
| --- | --- | --- | --- |
| Response to steroids | 4.92 | 1.57-17.33 | 0.009 |
| Use of neuro-muscular blockade | 0.38 | 0.02-4.05 | 0.45 |
| Use of proning | 2.64 | 0.82-9.23 | 0.11 |
| Fluid balance/weight on day of starting steroid | 1.00 | 0.99-1.00 | 0.37 |
| PELOD score | 0.92 | 0.85-1.00 | 0.06 |
| Immune compromise | 0.35 | 0.06-1.97 | 0.22 |
| OSI on day of starting steroids | 0.95 | 0.86-10.3 | 0.22 |
